# Morphological and molecular evidence indicate *Dendronotus primorjensis* is a valid species that has priority over *D. dudkai* (Nudibranchia)

**DOI:** 10.3897/zookeys.634.10231

**Published:** 2016-11-21

**Authors:** Tatiana A. Korshunova, Nadezhda P. Sanamyan, Alexander V. Martynov

**Affiliations:** 1 Koltzov Institute of Developmental Biology RAS, Vavilova Str. 26, 119334 Moscow Russia; 2 Kamchatka Branch of Pacific Geographical Institute FEB RAS, Partizanskaya Str. 6, 683000 Petropavlovsk-Kamchatsky Russia; 3 Zoological Museum of the Moscow State University, Bolshaya Nikitskaya Str. 6 125009 Moscow Russia

**Keywords:** Dendronotus, Gastropoda, Mollusca, Nudibranchia, new synonym

## Abstract

Morphological and molecular data of type material of the nudibranch mollusc *Dendronotus primorjensis* Martynov, Sanamyan, Korshunova, 2015 from the Sea of Japan are summarised and compared with those of *Dendronotus dudkai* Ekimova, Schepetov, Chichvarkhina, Chichvarkhin, 2016. The clear conclusion is that the latter is a junior synonym of *Dendronotus primorjensis*.

## Introduction

Martynov et al. (2015a, b) described the nudibranch *Dendronotus primorjensis* from the north-west part of the Sea of Japan. The original description of *Dendronotus primorjensis* provided numerous diagnostic features including external characters, detailed scanning electron microscopy images of the radula, as well as remarks about its molecular phylogenetic relationships. The format of that publication precluded publication of lengthy molecular analyses, even though they were performed. Subsequently, Ekimova et al. (2016) described *Dendronotus dudkai* as new from exactly the same region in which the type material of *Dendronotus primorjensis* was collected. Nevertheless Ekimova et al. (2016) considered *Dendronotus primorjensis* a *nomen dubium* and presented weak evidence of its invalidity, including allegations that the type material of *Dendronotus primorjensis* “are lost if ever existed” (Ekimova et al. 2016: 31). Original type material of *Dendronotus primorjensis* (holotype and paratype) has been stored in the Zoological Museum of Moscow State University since 2014.

In this publication analysis of combined molecular and morphological data of the holotype and paratype of *Dendronotus primorjensis* demonstrate that it is a valid species and has taxonomic priority over *Dendronotus dudkai*, published one year later.

## Material and Methods

### Type material collection

Type specimens of *Dendronotus primorjensis* (ZooBank: http://zoobank.org/2001DB85-2005-4E6F-8A21-F15F9068EC7D) have been described previously (Martynov et al. 2015a, b) and were used for both morphological and molecular examinations. The holotype of *Dendronotus primorjensis* (ZMMU Op-419) and the paratype (ZMMU Op-420) were collected in the Sea of Japan, Spokoinaya Bay, at the depth of 2–6.5 m (rocks and algae) on 25 Sept 2014, by T.A. Korshunova and A.V. Martynov using SCUBA diving. Photographs of the living animals were taken by T.A. Korshunova within a day of collection. In addition, staff members of the Diving Center Aquamax (Nakhodka) made additional photographic records immediately upon completion of the collecting dive. These photographs are permanently and publicly available since 30 September of 2014 (Diving Center Aquamax 2014). Afterwards, specimens were fixed in 75% ethanol for morphological study and in 99% ethanol for molecular investigations, and registered in the collection of the Zoological Museum of Moscow State University
(ZMMU) under the registration numbers Op-419 and Op-420.

### Morphological analysis

All specimens of *Dendronotus primorjensis* were examined with a stereomicroscope (MBS-9), a digital camera (Nikon D-90) with a set of extension rings (Kenko), and scanning electron microscope (CamScan Series II) for the original description. The pharynx of the preserved holotype *Dendronotus primorjensis* (ZMMU Op-419) was removed and processed with a weak solution of domestic bleach (NaOCl). The radula of the holotype was examined under SEM at the electron microscopy laboratory of the Biological Faculty of Moscow State University, and these were also published in the original description.

### Molecular analysis

Small pieces of foot tissue of both specimens of *Dendronotus primorjensis* (ZMMU:Op-419 and ZMMU:Op-420) were used for DNA extraction with Diatom™ DNA Prep 100 kit (Isogene Lab.) according to the producer’s protocols. Extracted DNA was used as a template for the amplification of partial sequences of the mitochondrial genes cytochrome *c* oxidase subunit I (COI) and 16S, and also the nuclear gene 28S (C1-C2 domain). The primers that were used for amplification are: LCO 1490 (GGTCAACAAATCATAAAGATATTGG, Folmer et al. 1994); HCO 2198 (TAAACTTCAGGGTGACCAAAAAATCA, Folmer et al. 1994); 16S arL (CGCCTGTTTAACAAAAACAT, Palumbi et al. 1991); 16S R (CCGRTYTGAACTCAGCTCACG, Puslednik and Serb 2008); 28S C1' (ACCCGCTGAATTTAAGCAT, Dayrat et al. 2001); and 28S C2 (TGAACTCTCTCTTCAAAGTTCTTTTC, Le et al. 1993). Polymerase chain reaction (PCR) amplifications were carried out in a 20-µL reaction volume, which included 4 µL of 5x Screen Mix (Eurogen Lab), 0.5 µL of each primer (10 µM stock), 1 µL of genomic DNA, and 14 µL of sterile water. The amplification of COI and 28S was performed with an initial denaturation for 1 min at 95°C, followed by 35 cycles of 15 sec at 95°C (denaturation), 15 sec at 45°C (annealing temperature), and 30 sec at 72°C, with a final extension of 7 min at 72°C. The 16S amplification began with an initial denaturation for 1 min at 95°C, followed by 40 cycles of 15 sec at 95°C (denaturation), 15 sec at 52°C (annealing temperature), and 30 sec at 72°C, with a final extension of 7 min at 72°C. Sequencing for both strands proceeded with the Big Dye v3.1 sequencing kit (Applied Biosystems). Sequencing reactions were analysed using an ABI 3500 Genetic Analyser (Applied Biosystems). Protein-coding sequences were translated into amino acids for confirmation of the alignment. Both sequences of *Dendronotus primorjensis* (ZMMU:Op-419 and ZMMU:Op-420) were deposited in GenBank. Original data and publicly available sequences were aligned with the MUSCLE (Edgar 2004) algorithm. For phylogenetic reconstruction 31 specimens were used. All of the species and their sequences are listed in Table 1.

**Table 1.** List of specimens used for phylogenetic analyses.

Species	Voucher	Locality	GenBank accession nos.		
			CO1	16S	28S
*Dendronotus primorjensis* Martynov et al., 2015 holotype	ZMMU:Op-419	Russia: Japan Sea	KX672010	KX672008	KX672006
*Dendronotus primorjensis* Martynov et al., 2015 paratype	ZMMU:Op-420	Russia: Japan Sea	KX672011	KX672009	KX672007
*Dendronotus dudkai* Ekimova et al., 2016	W195	Russia: Japan Sea	KT031811	KT031824	KT031841
*Dendronotus dudkai* Ekimova et al., 2016	W196	Russia: Japan Sea	KT031812	KT031825	KT031842
*Dendronotus dudkai* Ekimova et al., 2016	W197	Russia: Japan Sea	KT031813	KT031826	KT031843
*Dendronotus dudkai* Ekimova et al., 2016	W198	Russia: Japan Sea	KT031814	KT031827	KT031844
*Dendronotus dudkai* Ekimova et al., 2016	W199	Russia: Japan Sea	KT031815	KT031828	KT031845
*Dendronotus dudkai* Ekimova et al., 2016	W201	Russia: Japan Sea	KT031816	KT031829	KT031837
*Dendronotus dudkai* Ekimova et al., 2016	W202_1	Russia: Japan Sea	KT031817	KT031830	KT031838
*Dendronotus dudkai* Ekimova et al., 2016	W202_2	Russia: Japan Sea	KT031818	KT031831	KT031840
*Dendronotus dudkai* Ekimova et al., 2016	W203	Russia: Japan Sea	KT031819	KT031832	KT031839
*Dendronotus dalli* Bergh, 1879	ZMMU:Op-295	Russia: Kamchatka	KM397001	KM397083	KM397042
*Dendronotus dalli* Bergh, 1879	ZMMU:Op-330	Russia: Kamchatka	KM396999	KM397081	KM397040
*Dendronotus frondosus* (Ascanius, 1774)	ZMMU:Op-380	Norway	KM396976	KM397056	KM397017
*Dendronotus frondosus* (Ascanius, 1774)	ZMMU:Op-324	Russia: Barents Sea	KM396980	KM397062	KM397021
*Dendronotus kalikal* Ekimova et al., 2015	ZMMU:Op-284.3	Russia: Kamchatka	KM396988	KM397070	KM397029
*Dendronotus kalikal* Ekimova et al., 2015	ZMMU:Op-349.1	Russia: Kamchatka	KM396986	KM397068	KM397027
*Dendronotus kamchaticus* Ekimova et al., 2015	ZMMU:Op-246.2	Russia: Kamchatka	KM396989	KM397072	KM397030
*Dendronotus kamchaticus* Ekimova et al., 2015	ZMMU:Op-247.1	Russia: Kamchatka	KM396991	KM397073	KM397032
*Dendronotus lacteus* (W. Thompson, 1840)	ZMMU:Op-288	Russia: Barents Sea	KM396975	KM397059	KM397016
*Dendronotus lacteus* (W. Thompson, 1840)	ZMMU:Op-335	Russia: Barents Sea	KM396973	KM397057	KM397014
*Dendronotus niveus* Ekimova et al., 2015	ZMMU:Op-269	Russia: White Sea	KM396996	KM397078	KM397037
*Dendronotus niveus* Ekimova et al., 2015	ZMMU:Op-279	Russia: Barents Sea	KM396995	KM397077	KM397036
*Dendronotus patricki* Stout et al., 2011	SIO-BIC M12133	USA: California	HQ225828	HQ225829	–
*Dendronotus regius* Pola & Stout, 2008	CASIZ179492	Philippines	HM162708	HM162629	–
*Dendronotus robustus* Verrill, 1870	ZMMU:Op-343	Russia: Barents Sea	KM397002	KM397084	KM397043
*Dendronotus robustus* Verrill, 1870	ZMMU:Op-344	Russia: Barents Sea	KM397003	KM397085	KM397044
*Doto coronata* (Gmelin, 1791)	CASIZ176278	South Africa	HM162734	HM162657	–
*Doto koenneckeri* Lemche, 1976	CASIZ178247	Portugal	HM162735	HM162658	–
*Marionia arborescens* Bergh, 1890	CAS:177735	Philippines	KP226855	KP226859	–
*Tritonia challengeriana* Bergh, 1884	CASIZ171177	Atlantic Ocean: Bouvet Island	HM162718	HM162643	–

Two different phylogenetic methods, Bayesian Inference (BI) and Maximum Likelihood (ML) were used to infer evolutionary relationships. Separate analyses were conducted for the following data sets: resulting alignments are 641 bp for COI, 451 bp for 16S, 350 bp for 28S, and 1442 for the concatenated datasets. Evolutionary models for each data set were selected using MrModelTest 2.3 (Nylander et al. 2004) under the Akaike information criterion (Akaike 1974). Bayesian estimation of posterior probability was performed in MrBayes 3.2. Markov chains were sampled at intervals of 500 generations. Analysis was started with random starting trees and 10^7^ generations. Maximum likelihood-based phylogeny inference was performed in GARLI 2.0 (Zwickl 2006) with bootstrap in 1000 pseudo-replications. The program TRACER v1.6 was used to examine the convergence results. Additionally, Automatic Barcode Gap Discovery (ABGD) (Puillandre et al. 2012) was used to define species. The ABGD program is available from http://wwwabi.snv.jussieu.fr/public/abgd/abgdweb.html. COI and 16S FASTA alignments were analysed separately (excluding outgroups) using both proposed models: Jukes-Cantor (JC69) and Kimura (K80). The program Mega7 (Kumar et al. 2016) was used to calculate the uncorrected COI p-distances between all the sequences.

## Results

### Morphological characters of *Dendronotus primorjensis*

**Figure 1.**
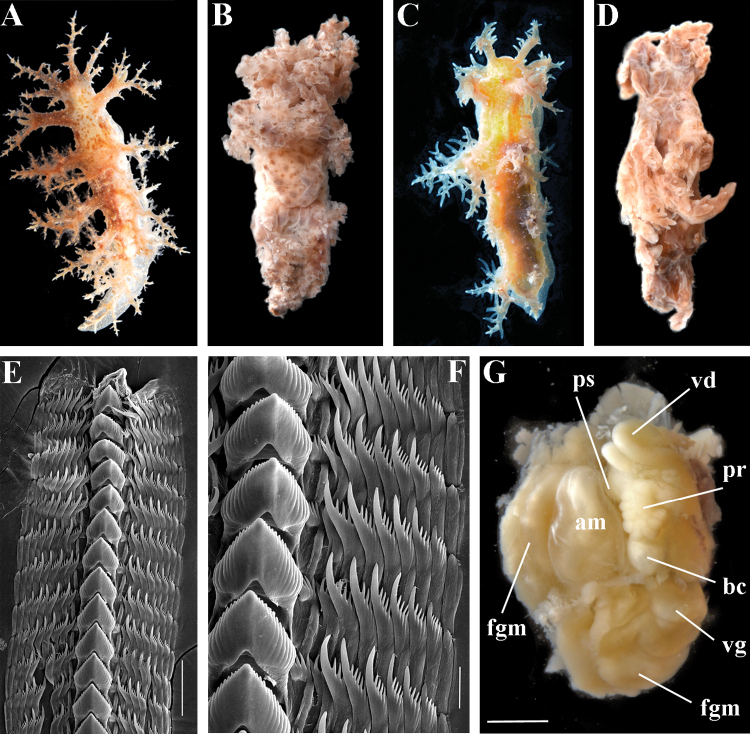
*Dendronotus primorjensis*, type material from Zoological Museum MSU: **A** holotype ZMMU Op-419, live, 35 mm in length, dorsal view **B** fixed holotype ZMMU Op-419 **C** paratype ZMMU Op-420, live, 21 mm in length **D** fixed paratype ZMMU Op-420 **E** radula of the holotype ZMMU Op-419, posterior part, SEM; **F** same, details **G** reproductive system of the holotype ZMMU Op-419. Abbreviations: **am** ampulla; **bc** bursa; **fgm** female gland mass; **pr** prostate; **ps** penial sheath; **vd** vas deferens; **vg** vagina. Scale bars **E** = 100 µm **F** = 30 µm **G** = 1 mm. Photos and SEM images by T.A. Korshunova and A.V. Martynov (Figures A, E, and F were published as part of the original description by Martynov et al. 2015a).

The holotype of *Dendronotus primorjensis* (ZMMU Op-419, Fig. 1A, B) and paratype (ZMMU Op-420, Fig. 1C, D) possess the following morphological features: body elongate, high, laterally compressed. Living length 21–35 mm. Oral veil narrow with 7-8 large branched appendages. Branched lateral papilla at middle of rhinophoral sheaths. Five appendages of rhinophoral stalks, 8–11 rhinophoral lamellae. 5–6 pairs of highly branched dorsolateral appendages, decreasing in size and branching towards tail. Digestive gland branches penetrate most dorsolateral appendages as well as rhinophoral sheaths. Dorsolateral appendages with long primary stalk, secondary branches, and elongate tertiary branches (Fig. 1A–D). Dorsal surface tuberculate. Foot narrow, rounded in front, narrowed towards tail. Reproductive opening placed laterally on right side at level of first pair of dorsolateral appendages. Anal opening on right side between first and second pairs of dorsolateral appendages. Colour non-uniformly reddish brown with few opaque white stripes between dorsolateral processes (holotype, Fig. 1A), more uniformly olive almost without white pigment (paratype, Fig. 1C), or almost lacking general pigmentation. Dorsum, dorsolateral appendages, and upper sides of foot with small scattered whitish and yellowish dots. 7–12 lip papillae. Dorsal processes of the jaws inclined posteriorly at approximately 47° to the longitudinal axis of the jaw body and 0.48 of its length; denticles present. Radular formula 37 × 8–9.1.9–8 (holotype) (Fig. 1E, F). Central tooth strong, distinctly denticulate in both anterior and posterior parts of radula, with up to 14 denticles (Fig. 1E, F) bearing deep furrows. Lateral teeth narrow, with relatively long curved cusp, bearing 3–6 distinct denticles (Fig. 1F). Outermost lateral teeth almost devoid of denticles. Reproductive system triaulic (Fig. 1G), ampulla twice folded. Prostate in holotype rounded, consists of no less than 19 alveolar glands. Distal part of vas deferens relatively short, entangled, and expanded to oval penial sheath and relatively long and curved conical penis. Vagina moderate in length. Bursa copulatrix large, rounded, elongated, seminal receptaculum placed distally (nomenclature of the seminal reservoirs according to Stout et al. 2011).

### Morphological characters of *Dendronotus dudkai*

These characters are taken directly from Ekimova et al. (2016: 35, 37, shortened):

“Body elongate, laterally compressed [the range of the lengths of specimens was not recorded in the description of *Dendronotus dudkai* by Ekimova et al. (2016), and only from the figure legends is it possible to estimate that length can be up to 28 mm]. Oral veil with 6–12 large, secondary branched cerata. 5–10 short lip papillae. Rhinophoral sheaths with long stalk and 4–5 crown secondary branched appendages. Lateral papillae moderate in size with small secondary branches. Rhinophores with 8–10 lamellae. 6–8 pairs of highly branched dorsolateral processes, size and degree of branching decrease towards the tail. Secondary branches long and rounded, tertiary branches short and sometimes pointed. General colour pattern varies from beige to dark-brown. Background colour translucent-white or light yellow. A lot of spots, stripes and dots on dorsal side of the body, cerata, rhinophoral sheath processes and upper parts of foot. Their colour varies from yellow to dark-brown. Some specimens covered with dots of golden or white opaque pigment. This pigment locates also in low body papillae and small tubercles. All specimens possess well-visible white stripes between pairs of cerata. Dorsal processes of jaws about 2.5 times shorter than jaw body. Inclined posteriorly at about 45°. Masticatory process about one-third as long as jaw body, slightly curved at base and become transparent and subulate posteriorly. Masticatory border with a single raw of denticles. Radula formula up to 32 × 7–8.1.7–8. Rachidian tooth bears 12–18 sharp denticles with thin furrows on both sides of the reduced cusp. Lateral teeth slightly curved, bear 4–8 well-defined denticles. Reproductive system triaulic. Ampulla wide and sinuous. Prostate concentric ring-shaped, consists of 12–14 oval alveolar glands. Distal part of vas deference winding expand into wide, muscular portion. Penis slightly curved. Oviduct connects through insemination duct into female gland complex. Vagina long, convoluted, rounded seminal receptaculum, small bursa copulatrix” [according to the updated nomenclature by Stout et al. 2011, receptaculum = bursa and vice versa].

### Phylogenetic analysis

Brief molecular results, including the genetic distances between *Dendronotus primorjensis* and closely related species were provided in two previous studies (Martynov et al. 2015a, b). In this study an extended molecular analysis is provided for a detailed comparison of the molecular data between *Dendronotus primorjensis* and *Dendronotus dudkai*.

**Figure 2.**
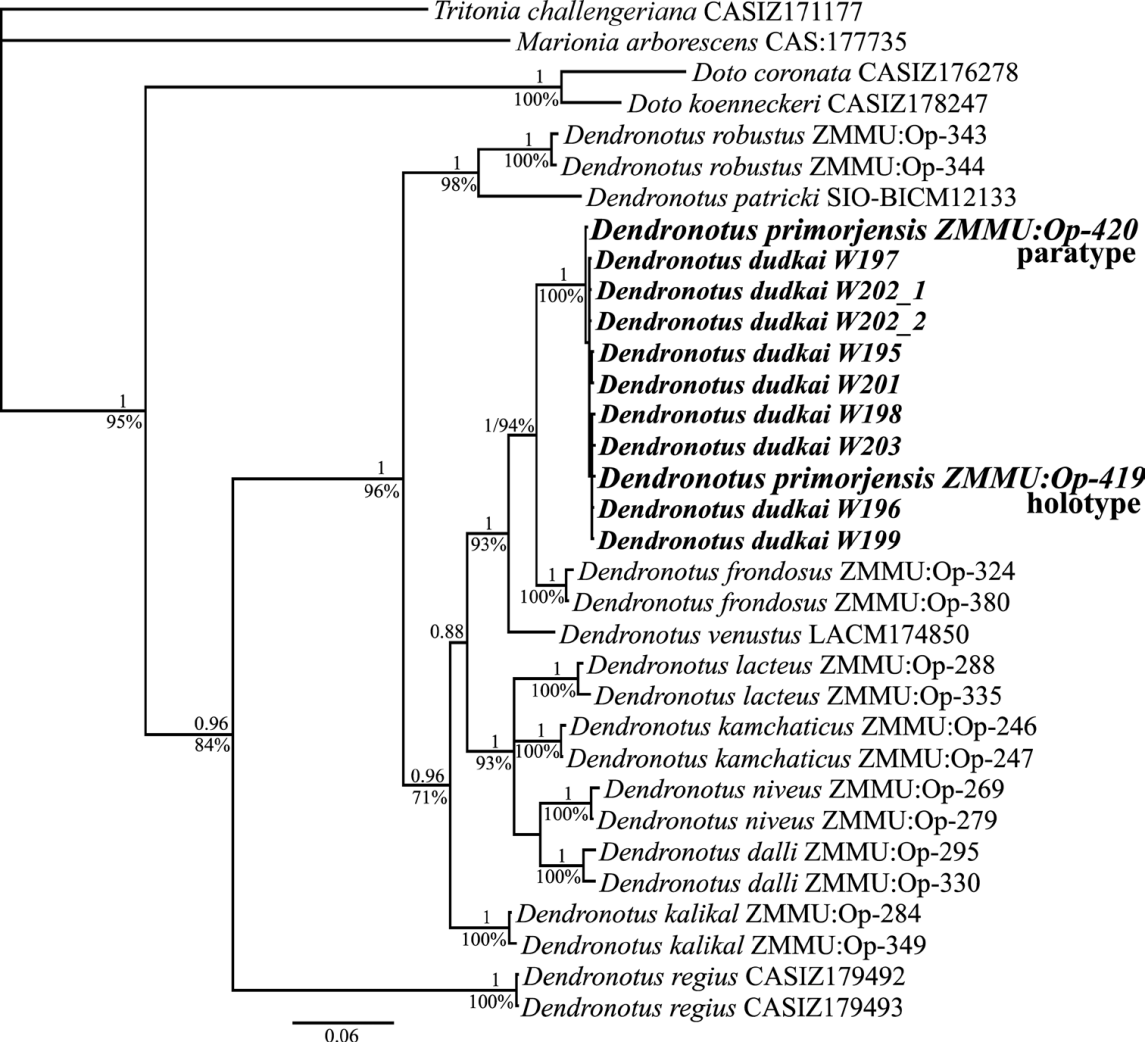
Phylogenetic tree based on combined molecular data (COI + 16S + 28S) represented by Bayesian Inference. Numbers above branches represent posterior probabilities from BI. Numbers below branches indicate bootstrap values for Maximum Likelihood.

Phylogenetic analyses were separately performed for COI, 16S, and 28S genes, and three concatenated nucleotide datasets from the holotype and paratype of *Dendronotus primorjensis*, available *Dendronotus* specimens from GenBank, and an outgroup consisting of four species of *Doto*, *Tritonia*, and *Marionia* (Table 1). Trees of both Bayesian Inference (BI) and Maximum Likelihood (ML) were used to infer phylogenetic trees. All single-gene trees as well as concatenated trees revealed very low divergence between *Dendronotus primorjensis* and *Dendronotus dudkai* specimens. A phylogenetic tree based on combined molecular data is represented in Figure 2. The combined data set including the three loci was presented in a sequence alignment of 1442 codon positions. The General Time Reversal model with invariant sites and gamma distribution (GTR+I+G) was selected as the best model for three nucleotide datasets. The topologies of phylogenetic trees inferred from two methods (ML and BI) and three datasets were identical. *Dendronotus primorjensis* and *Dendronotus dudkai* specimens are clustered in a single clade with maximum support (PP = 1, BS = 100%).

The ABGD analysis of the COI data set run with two different models revealed eleven potential species each:

*Dendronotus regius* (CASIZ179492, CASIZ179493);

*Dendronotus lacteus* (ZMMU:Op-288, ZMMU:Op-335);

*Dendronotus kamchaticus* (ZMMU:Op-246, ZMMU:Op-247);

*Dendronotus niveus* (ZMMU:Op-269, ZMMU:Op-279);

*Dendronotus dalli* (ZMMU:Op-295, ZMMU:Op-330);

*Dendronotus kalikal* (ZMMU:Op284, ZMMU:Op349);

*Dendronotus venustus* (LACM174850);

*Dendronotus primorjensis* (ZMMU:Op-419, ZMMU:Op-420) together with *Dendronotus dudkai* (W195, W196, W197, W198, W199, W201, W202_1, W202_2, W203);

*Dendronotus frondosus* (ZMMU:Op-380, ZMMU:Op-324);

*Dendronotus patricki* (SIO-BICM12133);

*Dendronotus robustus* (ZMMU:Op-343; ZMMU:Op-344).

The prior maximal distance ranged between 0.001 and 0.059.

The ABGD analysis of the 16S data set run with two different models revealed ten potential species each:

*Dendronotus regius* (CASIZ179493, CASIZ179492);

*Dendronotus robustus* (ZMMU: Op344, ZMMU:Op343);

*Dendronotus patricki* (SIO-BIC M12133);

*Dendronotus kalikal* (ZMMU:Op349, ZMMU:Op349);

*Dendronotus venustus* (LACM:174852.1);

*Dendronotus frondosus* (ZMMU:Op324, ZMMU:Op380) together with *Dendronotus primorjensis* (ZMMU:Op-419, ZMMU:Op-420) together with *Dendronotus dudkai* (W195, W196, W197, W198, W199, W201, W202_1, W202_2, W203);

*Dendronotus niveus* (ZMMU:Op269, ZMMU:Op279);

*Dendronotus lacteus* (ZMMU:Op288, ZMMU:Op335);

*Dendronotus kamchaticus* (ZMMU:Op247, ZMMU:Op246);

*Dendronotus dalli* (ZMMU:Op295, ZMMU:Op330).

The prior maximal distance ranged between 0.001 and 0.021.

Results for genetic distances between *Dendronotus primorjensis*, *Dendronotus frondosus*, *Dendronotus venustus*, *Dendronotus kalikal*, and *Dendronotus kamchaticus* have been described previously (Martynov et al. 2015b). Uncorrected COI p-distances between the holotype and paratype of *Dendronotus primorjensis* and other species of the genus *Dendronotus* are listed in Table 2. The COI p-distances of *Dendronotus primorjensis* – *Dendronotus dudkai* specimens range from 0 to 0.31%, indicating a strong overlap in the molecular dataset. Thus, the results unmistakeably indicate that *Dendronotus primorjensis* and *Dendronotus dudkai* belong to the same species, and *Dendronotus dudkai* is here regarded as a junior synonym.

**Table 2.** Uncorrected COI p-distances (%) between holotype and paratype of *Dendronotus primorjensis* and other species of the genus *Dendronotus*.

Species	*Dendronotus primorjensis* ZMMU:Op-419 holotype	*Dendronotus primorjensis* ZMMU:Op-420 paratype
*Dendronotus primorjensis* paratype	0.16	-
*Dendronotus dudkai* KT031811	0.16	0.31
*Dendronotus dudkai* KT031814	0.16	0.31
*Dendronotus dudkai* KT031816	0.16	0.31
*Dendronotus dudkai* KT031819	0	0.16
*Dendronotus dudkai* KT031812	0	0.16
*Dendronotus dudkai* KT031813	0	0.16
*Dendronotus dudkai* KT031815	0	0.16
*Dendronotus dudkai* KT031817	0	0.16
*Dendronotus dudkai* KT031818	0	0.16
*Dendronotus frondosus* KM396976	6.71	6.55
*Dendronotus venustus* HM162709	8.11	7.96
*Dendronotus kalikal* KM396986	11.2	11.4
*Dendronotus kamchaticus* KM396989	11.86	11.7
*Dendronotus albopunctatus* GQ292064	12.01	11.86
*Dendronotus niveus* KM396996	12.32	12.17
*Dendronotus dalli* KM397001	13.88	13.73
*Dendronotus lacteus* KM396975	14.51	14.35
*Dendronotus patricki* HQ225828	14.7	14.5
*Dendronotus robustus* KM397002	14.8	14.7
*Dendronotus regius* HM162708	16.8	16.7

## Discussion

According to the morphological and molecular data presented above there are no species-level differences between *Dendronotus primorjensis* (Figs 1 and 2; Table 2) and *Dendronotus dudkai* (Ekimova et al. 2016: 33–37). Molecular analysis robustly places the holotype of *Dendronotus primorjensis* inside the clade of *Dendronotus dudkai* (Fig. 2). Both the external and internal morphologies, as well as radular and reproductive features, do not demonstrate any significant differences between the two. Other differences between the original descriptions of *Dendronotus primorjensis* and *Dendronotus dudkai* are discussed below.

Since *Dendronotus frondosus* is a closely related species to *Dendronotus primorjensis* (= *Dendronotus dudkai* syn. n.)(Fig. 2) and, according to Ekimova et al. (2016), co-occurs with *Dendronotus primorjensis* in the Sea of Japan, it is important to discuss distinguishing morphological characters between these two species as well as their biogeographical patterns. In the first description of *Dendronotus primorjensis* “an absence of the large amount of the white pigment” was described as a potential distinguishing external character from *Dendronotus frondosus* and the eastern Pacific related species *Dendronotus venustus* (Martynov et al. 2015a: 60). This statement was challenged by Ekimova et al. (2016: 31, 35) who claim that “all specimens possess well-visible white stripes between pairs of cerata.” However, surprisingly, on the figures of *Dendronotus dudkai* (Ekimova et al. 2016: fig. 9A, C) no white pigment is visible, thus in full agreement with the data on both the holotype and paratype of *Dendronotus primorjensis* (Martynov et al. 2015: 60, Fig. 5A–G and present study, Fig. 1A, C). The colour patterns of *Dendronotus frondosus* and *Dendronotus venustus* vary considerably (Robilliard 1970; pers. obs.) and *Dendronotus primorjensis* is potentially expected to have similar variations. However, to date no specimens of *Dendronotus primorjensis* with really large amounts of white pigment between the dorsolateral appendages have been reported (as previously documented for *Dendronotus frondosus* and *Dendronotus venustus*).

Ekimova et al. (2016: 37) claim that “*Dendronotus frondosus* is differentiated from *Dendronotus dudkai* by the absence of denticles on the masticatory process of jaws” but this is in error: in Robilliard’s review on the genus *Dendronotus*, the presence of denticles has been indicated for *Dendronotus frondosus* *sensu lato* (“A small number of relatively large, black denticles adorns the masticatory margin”, Robilliard 1970: 443). Furthermore, since Robilliard’s study refers to a mixture of *Dendronotus frondosus* *s. str.* and *Dendronotus venustus* MacFarland, 1966 (but anyway not *Dendronotus primorjensis*) we have studied a topotype of *Dendronotus frondosus* *s. str.* from the North Atlantic (Norway) to clarify the situation. Species identity was confirmed using both morphological and molecular data: the topotype of *Dendronotus frondosus* *s. str.* demonstrated several small but distinct denticles on the jaws. In the original description of *Dendronotus venustus*, numerous denticles (between 27 and 40) on the masticatory processes were also described (MacFarland 1966: 277). Thus, the presence of denticles on the masticatory processes of the jaws cannot serve as a diagnostic character for *Dendronotus primorjensis* (= *Dendronotus dudkai* syn. n.) despite claims by Ekimova et al. (2016).

Instead, it is suggested here that the number of denticles on the central teeth is a better diagnostic, and is higher in *Dendronotus primorjensis* (commonly more than 12, in holotype 14, reported range 12–18) than in *Dendronotus frondosus* (commonly up to 12, rarely up to 14 denticles in the topotype specimens from North Atlantic, pers. obs., reported range 8–12). The original description of the *Dendronotus primorjensis* is accompanied by two detailed SEM images of the radula (see Martynov et al. 2015a: fig. 5). On these images up to 14 denticles of the central radular tooth can be recognized, whereas according to Ekimova et al. (2016), the central tooth of *Dendronotus frondosus* from the Sea of Japan bears a maximum of 12 denticles. Therefore, a larger number of denticles on the central teeth of *Dendronotus primorjensis* distinguishes it from the Japan Sea’s *Dendronotus frondosus*, but was not discussed by Ekimova et al (2016). No other species of *Dendronotus* with a similar radula have been so far reported from the Sea of Japan. The discussion of the origin of *Dendronotus frondosus* in the Sea of Japan in the Ekimova et al. (2016: 38–39) mainly concentrates on the idea that *Dendronotus frondosus* could be a natural amphiboreal species. However, the fact that the only Japan Sea *Dendronotus frondosus* specimens recorded in Ekimova et al. (2016) were found exclusively in the vicinity of the Institute of Marine Biology FEB RAS (Vladivostok) indicates a high probability of anthropogenic introduction of *Dendronotus frondosus*. On the contrary, *Dendronotus primorjenisis* (= *Dendronotus dudkai* syn. n.) has a broad distribution in the Sea of Japan.

The number of lobules present in the prostate of *Dendronotus dudkai* are described by Ekimova et al. (2016: 31) as being among the “most important diagnostic characters” for the genus *Dendronotus*. This is inaccurate, as Ekimova et al. (2016: 32) indicate 16–30 prostatic lobules for *Dendronotus frondosus*, whereas 12–14 lobules are described for *Dendronotus dudkai* (Ekimova et al. 2016: 37). However, the holotype of *Dendronotus primorjensis* possesses no less than 19 lobules in the prostate clearly implying that variability of numbers of prostatic lobules in the genus *Dendronotus* can be significant, and therefore cannot be species-diagnostic. The number of prostate lobules has recently been shown in Korshunova et al. (2016) to be an unreliable character in several *Dendronotus* species.

Unfortunately, the description of *Dendronotus dudkai* contains a number of problems that prevent the repetition of their results obtained by molecular phylogenetic analysis. Despite the presence of a molecular analysis that was based on “additional material” (i.e. non-type specimens) no molecular data is available for the type material. According to their Table 1 and “Type material” section (Ekimova et al. 2016:19–21, 33-35) five specimens were listed as type material for *Dendronotus dudkai*, but the molecular data for only one paratype (ZMMU Lc-40366) was registered at GenBank; however, this voucher number for this paratype of *Dendronotus dudkai* does not exist in GenBank: specimen W203 was registered at GenBank instead of specimen ZMMU Lc-40366, but specimen W203 is completely absent in the “Material” list or Table 1. In addition, no illustrations of the holotype of *Dendronotus dudkai* were provided nor were characters of the holotype mentioned in the description. Thus, the analysis of Ekimova et al. (2016) leads to the conclusion that there is no evidence that description of *Dendronotus dudkai* is based on the primary type, i.e. the holotype. According to the Recommendations 73A, 73B, and 73C of ICZN (1999) the “author who establishes a new nominal species-group taxon should designate its holotype in a way that will facilitate its subsequent recognition”, “an author should designate as holotype a specimen actually studied by him or her, not a specimen known to the author only from descriptions or illustrations in the literature”, and “an author who establishes a new nominal species-group taxon should publish at least the following data concerning the holotype, if they are relevant and known to the author, including: 73C.1. its size or the size of one or more relevant organs or parts.” Thus, the absence of the holotype’s morphological (and molecular data), and even its measurements in the original description of Ekimova et al. (2016) do not allow anyone to recognize it.

## Conclusions

The morphological and molecular data provided in this publication and two previous studies (Martynov et al. 2015 a, b) conclusively demonstrate that *Dendronotus primorjensis* is a valid species as per current ICZN regulations (ICZN 1999). The integrative taxonomy (Dayrat 2005) presented here indicate that *Dendronotus primorjensis* and *Dendronotus dudkai* are one and the same species, and that *Dendronotus primorjensis* has taxonomic priority over *Dendronotus dudkai* published one year later. The species epithet *Dendronotus dudkai* is therefore to be considered a junior synonym of *Dendronotus primorjensis*.
